# A hybrid approach to comparing parallel-group and stepped-wedge cluster-randomized trials with a continuous primary outcome when there is uncertainty in the intra-cluster correlation

**DOI:** 10.1177/17407745221123507

**Published:** 2022-09-09

**Authors:** Samuel K Sarkodie, James MS Wason, Michael J Grayling

**Affiliations:** Population Health Sciences Institute, Newcastle University, Newcastle upon Tyne, UK

**Keywords:** Assurance, Bayesian-frequentist, expected power, hybrid design, intra-class correlation

## Abstract

**Background/Aims:**

To evaluate how uncertainty in the intra-cluster correlation impacts whether a parallel-group or stepped-wedge cluster-randomized trial design is more efficient in terms of the required sample size, in the case of cross-sectional stepped-wedge cluster-randomized trials and continuous outcome data.

**Methods:**

We motivate our work by reviewing how the intra-cluster correlation and standard deviation were justified in 54 health technology assessment reports on cluster-randomized trials. To enable uncertainty at the design stage to be incorporated into the design specification, we then describe how sample size calculation can be performed for cluster- randomized trials in the ‘hybrid’ framework, which places priors on design parameters and controls the expected power in place of the conventional frequentist power. Comparison of the parallel-group and stepped-wedge cluster-randomized trial designs is conducted by placing Beta and truncated Normal priors on the intra-cluster correlation, and a Gamma prior on the standard deviation.

**Results:**

Many Health Technology Assessment reports did not adhere to the Consolidated Standards of Reporting Trials guideline of indicating the uncertainty around the assumed intra-cluster correlation, while others did not justify the assumed intra-cluster correlation or standard deviation. Even for a prior intra-cluster correlation distribution with a small mode, moderate prior densities on high intra-cluster correlation values can lead to a stepped-wedge cluster-randomized trial being more efficient because of the degree to which a stepped-wedge cluster-randomized trial is more efficient for high intra-cluster correlations. With careful specification of the priors, the designs in the hybrid framework can become more robust to, for example, an unexpectedly large value of the outcome variance.

**Conclusion:**

When there is difficulty obtaining a reliable value for the intra-cluster correlation to assume at the design stage, the proposed methodology offers an appealing approach to sample size calculation. Often, uncertainty in the intra-cluster correlation will mean a stepped-wedge cluster-randomized trial is more efficient than a parallel-group cluster-randomized trial design.

## Introduction

A cluster-randomized trial (CRT) randomizes groups of individuals (or ‘clusters’, e.g. defined by a hospital or school), rather than individuals themselves.^1^ Although many CRT designs are available, this article focuses on parallel-group (PG) and stepped-wedge (SW) designs. We refer the reader to Hemming and Taljaard^2^ for an extensive discussion and comparison of these two designs. For either design, an issue in practice is specifying certain nuisance parameters at the design stage for sample size estimation. Misspecification of these parameters has considerable implications on the power^3^ and, by extension, the validity of the trial.^4^

Specifically, in CRTs, an essential component of sample size determination is the intra-class correlation (ICC), which quantifies the degree of similarity between individuals within a cluster. In practice, the ICC is specified based on past data or studies,^5,6^ pilot studies^7,8^ or by choosing a sufficiently ‘conservative’ value. The significance of using precise estimates of the ICC or accounting for its impression during sample size computation is discussed by Ukoumunne et al.^9^ This recommendation was amplified in the Consolidated Standards of Reporting Trials (CONSORT) guidelines for CRTs by encouraging the reporting of ICC values and their uncertainty.^10^ However, a systematic review of ICC reporting by Han et al.^11^ found that only 26% of 281 CRTs reported the actual estimated values of the ICC, indicating a low adherence to the CONSORT principles. A recent review of publicly funded trials in the United Kingdom also found that 42% of the ICCs for the analysed primary outcomes were not reported, 12% of the studies did not report the ICC at all, while the remaining reported it via point estimates.^12^ Even in trials where the ICC values are available, other criteria such as the number of clusters, average cluster size, subjects, setting, stratification, and outcomes needed to establish if the ICC is suitable for the study being designed are often not published.^13^ Thus, the paucity of ICC values and the likelihood of estimate misspecification may be negatively impacting the success of many trials.

A possible solution to the issues around specifying the ICC or other design parameters could be a ‘hybrid’ (sometimes called ‘Bayesian-frequentist’) approach that places a prior on these parameters. Using a hybrid approach, we incorporate uncertainty in particular parameters within the trial design, mitigate the risk of overly optimistic power calculations, and satisfy most regulatory agency guidelines by maintaining a frequentist framework for the final analysis.^14^ Such approaches have received significant attention in the context of individually randomized trials (see, e.g. Kunzmann et al.^15^), but limited attention for CRTs. In CRTs, previous work on incorporating uncertainty about the ICC has focused on how to formally quantify uncertainty based on estimates from past studies, compute an associated power distribution, and use an informative prior for the ICC in a trial’s analysis.^16,17^ Jones et al.^18^ also discussed the application of Bayesian methods to CRTs based on a systematic review. The most relevant work to CRTs designed within the hybrid framework is that of Lewis and Julious^7^ who, based on results from Ukoumunne,^19^ leveraged confidence intervals characterizing a plausible range for the ICC to incorporate uncertainty in its value into the sample size calculation. This is similar to a hybrid approach but does not associate a particular prior density to each possible ICC value.

We, therefore, describe how to determine the minimal sample size required to achieve a desired expected power (EP), one of the quantities primarily controlled in the hybrid literature.^14^ We do this for a setting in which a prior is placed not only on the ICC but also on the standard deviation (SD), for which there may also be substantial uncertainty at the design stage. We then present case studies of PG- and SW-CRTs which assumed ‘conservative’ values for the ICC in their sample size determination; the required sample sizes from the conventional frequentist approach are compared against the sample sizes obtained when a prior is placed on the SD and the ICC. We then provide a critical evaluation of how placing a prior on the ICC impacts whether a PG- or SW-CRT design is more efficient, extending previous comparisons under a fixed ICC, such as those by Hemming and Taljaard^20^ and Woertman et al.^21^ To provide further context and motivation for our work, we also review a selection of published health technology assessment reports that utilized a CRT to determine how they justified their assumed ICC.

## Methods

### Review of published CRTs

The following search was run on PubMed on 08/01/21: (((‘Health technology assessment’ (Journal) OR (‘Health technology assessment reports’ (Journal) OR (‘Health technology assessment (Winchester, England)’ (Journal) AND (‘cluster’). The 54 returned articles were equally allocated between the three authors (S.K.S., M.J.G., and J.M.S.W.) to determine whether they related to the report of a completed CRT. For those articles that did, information was then extracted on the justification given for the assumed ICC and the SD, along with the assumed value of the ICC.

### CRTs designed within the hybrid framework

We seek to describe how sample size calculation for a PG- or SW-CRT can be performed in a hybrid framework. For brevity, we restrict our attention throughout to the case where the outcome data are assumed to be normally distributed. For the SW-CRT component, we focus on the case of a ‘cross-sectional’ design but comment in the Discussion section on extensions to this.

We assume the following model will be used for analysis of PG-CRTs

$$Y_{\mathrm{ij}}=\mu_{C}+X_{j}\mu +c_{j}+e_{\mathrm{ij}}$$

where $Y_{\mathrm{ij}}$ is the outcome from patient i=1,…,N (thus we assume N participants per cluster) in cluster j=1,…,C (thus we assume C clusters); $\mu_{C}$ is an intercept term or the mean outcome of the control group; $X_{j}=1$ if cluster j is allocated to the experimental arm and $X_{j}=0$ otherwise; μ captures the effect of the intervention relative to the control; $c_{j}\;\sim \;N(0,\sigma_{c}^{2})$ is a random effect for cluster j, which allows for a non-zero correlation between outcomes from participants within the same cluster; and $e_{\mathrm{ij}}\;\sim \;N(0,\sigma_{e}^{2})$ is the individual-level error.

As proposed by Hussey and Hughes,^22^ we extend this for SW-CRTs to

$$Y_{\mathrm{ijk}}=\mu_{C}+\beta_{j}+X_{\mathrm{jk}}\mu +c_{j}+e_{\mathrm{ijk}}$$

where $Y_{\mathrm{ijk}}$ is the outcome from patient i=1,…,n (thus we assume n participants per cluster-period) in cluster j=1,…,C, in time period k=1,…,T (thus we assume T time periods; which means there are N=nT measurements per cluster in total); $X_{\mathrm{jk}}=1$ if cluster j is allocated to the experimental arm in time period k and $X_{\mathrm{jk}}=0$ otherwise; and $\beta_{j}$ is a fixed effect for period j ( $\beta_{1}=0$ for identifiability). Due to the sequential roll out of the intervention in the SW-CRT, the model adjusts for the time period of collection. All other parameters are interpreted as above. Note that the sample sizes of the PG- and SW-CRT designs are both NC, with N=nT in the SW-CRT case. For the sensitivity analysis section of our results, we use the classical frequentist sample size equations (Appendix I in the supplemental material). We comment in the Discussion section on extensions to more complex analysis models for SW-CRTs.

In both instances the ICC is defined as $\rho =\sigma_{c}^{2}/(\sigma_{c}^{2}+\sigma_{e}^{2})=\sigma_{c}^{2}/\sigma^{2}$. This is the ratio of the variation between the clusters ( $\sigma_{c}^{2}$) to the total (between and within cluster) variation ( $\sigma^{2}$).

We assume our interest lies in testing that there is no positive treatment effect in the intervention arm, thus $H_{0}:\mu \leq 0$. We perform a test for $H_{0}$ using the test statistic $Z=\hat{\mu }/\sqrt{\mathrm{Var}(\hat{\mu })}$, rejecting $H_{0}$ when $Z\;>\;z_{1-\alpha }$, with α the desired type-I error-rate. Specifying a formula for the EP is thus dependent on knowing the variance of $\hat{\mu }$ for a given design. In the PG-CRT case, assuming 1:1 allocation of clusters to the two treatment conditions, it is well known that

(1) $$\mathrm{Var}\left(\hat{\mu }\right)=\frac{4\left\{1+\left(n-1\right)\rho \right\}\sigma^{2}}{\mathrm{Cn}}$$

Thus, the probability $H_{0}$ is rejected for a PG-CRT design (i.e. the frequentist power) is

(2) $$\Phi \left[\mu \sqrt{\frac{\mathrm{Cn}}{4\left\{1+\left(n-1\right)\rho \right\}\sigma^{2}}}-z_{1-\alpha }\right]$$

Similarly, it can be shown that for an SW-CRT^22,23^ that

(3) $$\mathrm{Var}\left(\hat{\mu }\right)=\frac{C\sigma^{2}\left(1-\rho \right)\left[1+\rho \left(\mathrm{nT}-1\right)\right]}{n\left\{\left[1+\rho \left(\mathrm{nT}-1\right)\right]\left(\mathrm{CU}-W\right)+n\rho \left(U^{2}-\mathrm{CV}\right)\right\}}$$

*where*

$$\begin{array}{c} U=\sum_{\mathrm{jk}}X_{\mathrm{jk}},\;W=\sum_{k}{\left(\sum_{j}X_{\mathrm{jk}}\;\right)}^{2},\\ \;V=\sum_{j}{\left(\sum_{k}X_{\mathrm{jk}}\right)}^{2} \end{array}$$

Thus, for an SW-CRT, the probability H_o_ rejected is

(4) $$\Phi \left[\mu \sqrt{\frac{n\left\{\left[1+\rho \left(nT-1\right)\right]\left(CU-W\right)+n\rho \left(U^{2}-CV\right)\right\}}{C\sigma^{2}\left(1-\rho \right)\left[1+\rho \left(nT-1\right)\right]}}-z_{1-\alpha }\right]$$

We denote the probability of rejecting $H_{0}$ for both designs by P(μ,n,X,α,β,σ,ρ). The parameter X is the matrix of binary treatment indicators; C×1 in the case of a PG-CRT and C×T for an SW-CRT. In the frequentist framework, a target difference μ=δ is allocated, and the study is designed to ensure power is at least 100(1−β)% in this instance, i.e. P(δ,n,X,α,σ,ρ)≥1−β. Here, β is the nominated type-II error-rate. Thus, in a conventional power calculation, the parameters ρ and σ take fixed specified values. As discussed, this negates consideration of any uncertainty in their nominated values. This can be addressed in a hybrid framework by placing a prior on the SD $\left(\psi_{\mathrm{SD}}(\sigma |\theta_{\mathrm{SD}})\right)$ and the ICC $\rho \;(\psi_{\mathrm{ICC}}(\rho |\theta_{\mathrm{ICC}}))$), allowing us to capture uncertainty in their values. Here, $\theta_{\mathrm{SD}}$ and $\theta_{\mathrm{ICC}}$ give parameters which describe the shape of the prior densities (e.g. its mean value and variance around this). We discuss specific choices for these priors later. Note that the use of the word ‘prior’ here may cause some confusion; $\psi_{\mathrm{SD}}$ and $\psi_{\mathrm{ICC}}$ capture the (prior beliefs about the) relative likelihood of different values of σ and ρ, they are not ‘priors’ in the fully Bayesian sense of the word (i.e. they will not be updated to posterior distributions).

In the hybrid framework, the usual frequentist power requirement is replaced by consideration of the value of the EP. The EP is a weighted average of the probability $H_{0}$ is rejected, with the weighting performed using $\psi_{\mathrm{SD}}$ and $\psi_{\mathrm{ICC}}$. Precisely, the EP for a PG- or SW-CRT is given by

(5) $$\begin{array}{c} \mathrm{EP}\left(n,C\right)=\int_{0}^{\infty }\int_{0}^{1}P\left(\delta ,n,X,\alpha ,\sigma ,\rho \right)\psi_{\mathrm{SD}}\\ \;\left(\sigma |\theta_{\mathrm{SD}}\right)\psi_{\mathrm{ICC}}\left(\rho |\theta_{\mathrm{ICC}}\right)d\rho d\sigma \end{array}$$

We explicitly list the EP as a function of n and C to reflect the fact that sample size calculation is often performed for CRTs by varying one or both of the parameters n and C. Computing a sample size in the hybrid framework then amounts to ensuring EP(n,C)≥1−γ, by suitable choice of n or C through a numerical search. Here, γ need not be equal to the value of β in the traditional frequentist framework, though this is a pragmatic and often assumed approach in the hybrid literature; we will therefore set γ=β throughout.

Later, to focus on a more specific question of interest, we also consider the scenario in which a prior is placed only on the ICC. In this case, Equation (5) above reduces to

(6) $$\mathrm{EP}\left(n,C\right)=\int_{0}^{1}P\left(\delta ,n,X,\alpha ,\sigma ,\rho \right)\psi_{\mathrm{ICC}}\left(\rho |\theta_{\mathrm{ICC}}\right)d\rho$$

### Choice of priors for the intra-cluster correlation and variance

What remains to be explained is logical choices for the priors $\psi_{\mathrm{SD}}$ and $\psi_{\mathrm{ICC}}$. We highlight that as these priors are not priors in the usual Bayesian sense of the word (i.e. they are not updated to posteriors), there are less logical restrictions on the distributional form of the priors to adopt. For the ICC, we may reasonably choose any distribution with support [0,1] and for the SD any distribution with support (0,∞). If the resultant values of, for example, $\psi_{\mathrm{ICC}}(\rho |\theta_{\mathrm{ICC}})$ are similar across ρ for two priors formed via different distributions, the resultant EPs should also be similar. For this reason, our choices below are not unique ones, nor should they be considered best practice; the best distribution for a particular trial will be one that results in prior densities most accurately reflecting beliefs about that parameter.

We explore normal and non-normal priors for the ICC and assess how they impact design. As in Turner et al.’s study,^16^ we first assume a truncated normal distribution is used for the ICC, truncated on [0,1]. We denote a prior assuming particular mean (m) and variance $\left(s^{2}\right)$ values for the original untruncated normal distribution by $\mathrm{TN}(0,1,m,s^{2})$. We then have

$$\psi_{\mathrm{ICC}}\left\{\rho |\left(m,s\right)\right\}=\frac{\phi \left(\frac{\rho -m}{s}\right)}{s\left\{\Phi \left(u\right)-\Phi \left(l\right)\right\}}$$

where l=(0−m)/s and u=(1−m)/s. Note that the mean and variance of $\psi_{\mathrm{ICC}}$ are then

$$\mathrm{Mean}=m+s\frac{\phi \left(u\right)-\phi \left(l\right)}{\Phi \left(u\right)-\Phi \left(l\right)}$$

$$\mathrm{Variance}=s^{2}\left[1+\frac{l\phi \left(l\right)-u\phi \left(u\right)}{\Phi \left(u\right)-\Phi \left(l\right)}-{\left\{\frac{\phi \left(l\right)-\phi \left(u\right)}{\Phi \left(u\right)-\Phi \left(l\;\right)}\right\}}^{2}\right]$$

In practice, the values of m and s could either be formed using methodology such as that provided by Turner et al.^17^ or elicited based on expert opinion.

Next, we assume a beta prior for the ICC since its support [0,1] is consistent with the range of the ICC. If we denote the prior by Beta(a,b), then

$$\psi_{\mathrm{ICC}}\left\{\rho |\left(a,\;b\right)\right\}=\frac{x^{a-1}{\left(x-1\right)}^{b-1}}{B\left(a,b\right)},\;a,b>0$$

where B is the Beta function. This prior has mean and variance given by

$$\mathrm{Mean}=\;\frac{a}{a+b}$$

$$\mathrm{Variance}=\frac{\mathrm{ab}}{{\left(a+b\right)}^{2}\left(a+b+1\right)}$$

Regarding the prior for the SD $\psi_{\mathrm{SD}}$, a convenient form in practice may be a Gamma distribution since this has support (0,∞). If we denote this by Gamma(k,θ), we have

$$\psi_{\mathrm{SD}}\left\{\sigma |\left(k,\;\theta \right)\right\}=\frac{\theta^{k}}{\Gamma \left(k\right)}\sigma^{k-1}e^{-\theta \sigma },\;\sigma \geq 0$$

which has

$$\mathrm{Mean}=\;\frac{k}{\theta }$$

$$\mathrm{Variance}=\frac{k}{\theta^{2}\;}.$$

### Motivating examples

We motivate assumed parameters for PG-CRT examples based on Surr et al.,^24^ a PG-CRT that sought to use Dementia Care Mapping to reduce agitation in care home residents with dementia. Hence, agitation at 16 months was the primary outcome, measured by the Cohen–Mansfield Agitation Inventory. This study was powered at 90% (β=0.1) with a 2.5% one-sided significance level (α=0.025) to detect a clinically important difference of 3 points (δ=3) with an SD of 7.5 points, (σ=7.5). An ICC of ρ=0.1 was assumed, leading to 50 care homes (C=50) being recruited with n=11 participants per cluster.

We use O’Grady et al.^25^ as motivation for assumed parameter values in SW-CRT examples. This SW-CRT aimed to implement a model that would improve outpatient substance use disorder treatment outcomes. The design had T=7 time periods, randomizing five clinics to begin the intervention in each of time periods 2 through 7 (i.e. C=30). The assumption was that there would be n=132 participants per clinic per time period. The study was powered at 80% (β=0.2) for α=0.005 and a clinically important difference of δ=0.0278. It assumed ρ=0.2 and σ=0.426.

### Software

Code to reproduce our results is available from https://github.com/sks2023/article_codes.

## Results

### Historical justifications for the intra-cluster correlation

Table 1 presents a summary, selected at random, of previous stated approaches to specifying the ICC from the reviewed Health Technology Assessment trials. The complete extracted data set is available in Supplementary File 1.

**Table 1.** Data extracted on assumed ICC values, SD, and their justification is given, for a random selection of reviewed Health Technology Assessment trials.

Reference	Assumed ICC	Justification for the assumed ICC/CV	Justification for the assumed SD
Surr et al.^24^	0.1	Conservative value based on a previous trial	Previous study
Heller et al.^26^	0.05	Assumed a value common in trial setting	None provided
Snooks et al.^27^	0.002	Conservative value based on a previous trial	Previous study
Sackley et al.^28^	0.4	Conservative value based on previous CRTs	Previous study
Richards et al.^29^	0.06	Based on a pilot study	None provided
Campbell et al.^30^	0.05	Based on a previous study and previous review work	Previous study
Stallard et al.^31^	0.025	Based on a pilot study	Pilot study
Forster et al.^32^	0.05	Based on previous research showing it was aconservative choice	None provided
Harris et al.^33^	0.5	None provided	None provided
MacArthur et al.^34^	0.01	Based on a previous study	Previous study

ICC: intra-cluster correlation, CV: coefficient of variation, SD: standard deviation.

As a first step in our review, 37% (20/54) of the papers were excluded as they did not meet the criteria for a CRT. Of the 34 papers that met the inclusion criteria, 21% had binary outcomes, while 79% had continuous outcomes. One CRT trial provided neither an assumed nor observed ICC value. The remaining 33 trials had similarities in how the ICC or coefficient of variation values were selected. Generally, a ‘conservative’ value was often assumed (29%), or it was stated that the ICC was based on a pilot study (12%) or a previous study in a similar setting (47%), while 12% of the trials provided no justification for their assumed ICC.

Unsurprisingly, none of the trials incorporated uncertainty around the values of the ICC by assuming a prior distribution or confidence interval for these parameters. However, few stated the power for a selection of point ICC values (see for example, Campbell et al.^30^), which was particularly surprising given the frequency with which the ICC was evidently not well understood at the design stage. In all, it is clear that in many trials there was uncertainty present in a suitable value of the ICC to assume during sample size calculation. Finally, we note that the assumed values for the ICC were positively skewed on [0.002, 0.5] with a median of 0.05; we return to this later when discussing our findings on the relative efficiency of PG- and SW-CRT designs.

### Example trials designed within the hybrid framework

**Figure 1.**
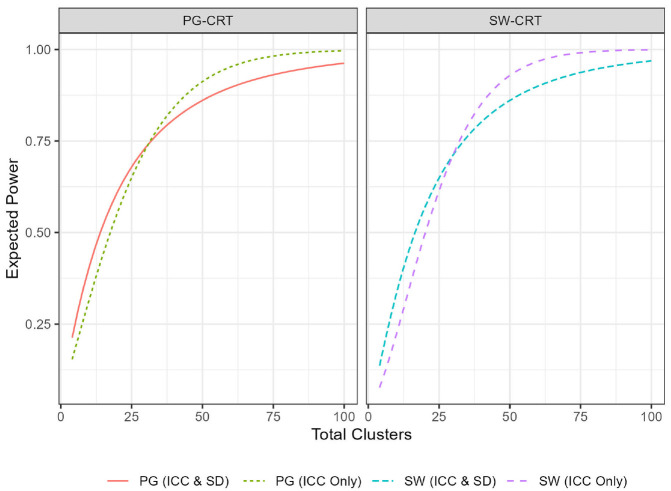
Plot of the expected power of several designs, as a function of the total number of clusters, C. The fixed parameter assumptions for PG-CRT were: α=0.025,δ=3,n=11; while SW-CRT assumed: α=0.005,δ=0.028,n=132,T=7. For the ICC only lines, σ=7.5 was assumed for the PG-CRT and σ=0.426 was assumed for the SW-CRT.

First, we provide a simple example of how the EP varies in the hybrid framework as a function of the number of clusters (Figure 1). We illustrate this relationship under two scenarios: (a) when a prior is placed only on the ICC while holding all other parameters from the motivating examples fixed, and (b) when priors are placed on both the ICC and the SD while holding all other parameters from the motivating examples fixed. Parameters for the priors (PG: $\psi_{\mathrm{ICC}}\;\sim \;\mathrm{TN}(0,1,0.1,0.01^{2})$, $\psi_{\mathrm{SD}}\;\sim \;\mathrm{Gamma}(75,10)$; SW: $\psi_{\mathrm{ICC}}\;\sim \;\mathrm{TN}(0,1,0.2,0.01^{2})$, and $\psi_{\mathrm{SD}}\;\sim \;\mathrm{Gamma}(17.32,40)$ were selected such that the mode of the prior is always equal to the point estimate assumed in the motivating example. In what follows, we term priors whose mode matches the corresponding frequentist assumptions as ‘correctly specified priors’.

Similar to the classical frequentist power, the EP increases as the number of clusters increases. Thus, like in a traditional sample size calculation, trials designed within the hybrid framework would simply require determination of the minimal number of clusters required to achieve the desired EP. Note that the EP, like the frequentist power, approaches 1 as the number of clusters is made very large. For both designs, having a prior only on the ICC resolves the EP curve to 1 more quickly, as including a prior on the SD incorporates consideration of power for large values of σ, which will be low. While the prior on the SD affects both designs in terms of the EP, the PG-CRT appears to be less affected. This implies that the EP of a design, and hence the required sample size, may be highly dependent on the robustness with which each design (PG vs SW) handles the prior weights.

**Figure 2.**
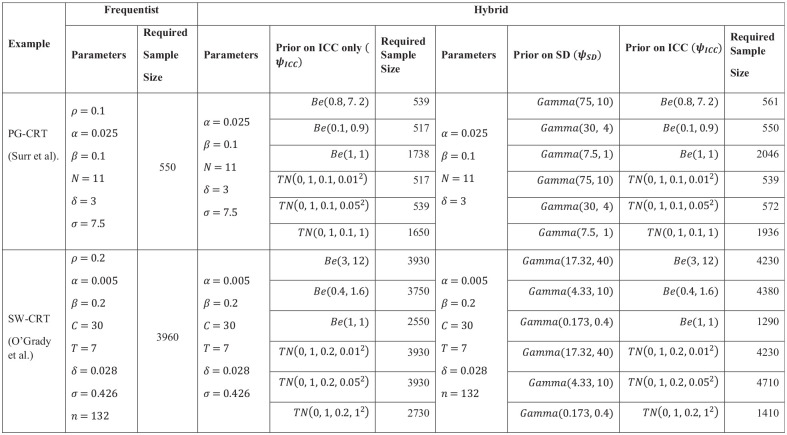
Comparison between the frequentist and hybrid approaches, for example, parameters motivated by Surr et al. (PG-CRT) and O’Grady et al. (SW-CRT); priors correctly specified.

**Figure 3.**
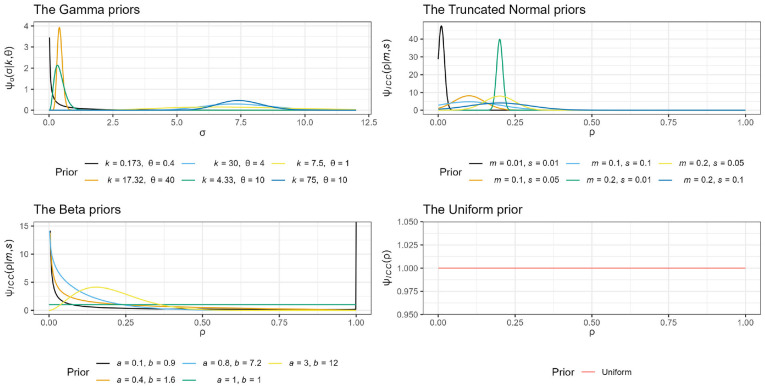
Plot of the Gamma, truncated Normal, Beta, and Uniform correctly specified priors.

Next, to expand on the above, we compare the two approaches (frequentist and hybrid) to sample size determination in CRTs in more depth, discussing the implications of choosing (a) a particular framework and (b) particular priors in the trials designed within the hybrid framework. For a fairer comparison, we compute required sample sizes based on control of the EP to the same level as that in the frequentist framework (i.e. γ=β) and employ correctly specified priors. This then leaves free choice of the variance of the priors. We therefore demonstrate how a ‘small’ ,‘moderate’, and ‘large’ prior variance affects the sample size required to achieve a desired EP for each design. In considering Beta and Truncated Normal priors for the ICC, we choose matching variances, so that a fairer comparison can be made between using a Beta or Truncated Normal distribution. Required sample sizes in the frequentist and hybrid frameworks for a selection of possible priors are presented in Figure 2 and a plot of all utilized priors is given in Figure 3.

When priors are correctly assigned to both the ICC and the SD, the number of participants required to achieve the desired EP is often higher than when a prior is correctly assigned to only the ICC. As discussed above in relation to Figure 1, the magnitude of the increase or decrease is determined by the variance of the prior. In particular, a small prior variance in the Truncated Normal and Gamma priors was the only scenario where the sample size under the hybrid approach was smaller than frequentist approach for the PG-CRT design. It is worth noting that while large prior variance on the PG-CRT designs requires more participants to achieve its EP, the SW-CRT design required less participants, compared to the frequentist framework.

We observe also that when a correctly specified prior is placed only on the ICC, and this prior has small or moderate variance, the hybrid approach for both CRT designs requires a smaller number of participants than the frequentist framework.

These findings highlight the sensitivity of the PG-CRT to variability in the ICC and the SW-CRT design’s known efficiency for higher ICC values. Specifically, a high ICC means that the clusters themselves are responsible for most of the outcome variance; hence the within-cluster comparisons facilitated by an SW-CRT become a rich source of information. A consequence is that, perhaps counterintuitively, incorporating larger uncertainty can lower the required sample size for an SW-CRT compared to a frequentist approach.

The choice of prior distribution and the level of uncertainty arising from its weightings are critical in the hybrid framework. For example, the uniform prior (Be(1,1)), increases the sample size under the PG-CRT relative to the SW-CRT. We later discuss the implications of such priors. Unsurprisingly, when a Beta or Truncated Normal distribution with similar densities are used as the prior, the resultant required sample size is similar. Where differences arise is when the desire for a small prior mode results in a Beta distribution with an undefined density at zero. In some settings, it may be the case that an extremely small ICC is a reasonable assumption. In general, though, it is this reason (along with non-statistician’s greater familiarity with the Normal distribution) that we prefer the use of a Truncated Normal prior for the ICC.

### Comparison of the EP provided by PG- and SW-CRT designs

To conclude, we include an important comparison of the EP provided by PG-CRT and SW-CRT designs when a prior is placed only on the ICC. This then serves to extend previous comparisons of which design is more efficient to the case where there is uncertainty in the ICC’s value.

The EP is now dependent on the assumed number of clusters ( C), the number of measurements per cluster ( n and nT for the PG- and SW-CRT designs respectively), the number of time periods in the SW-CRT design ( T), the standardized effect size ( δ/σ), as well as the assumed prior parameters m and s.

**Figure 4.**
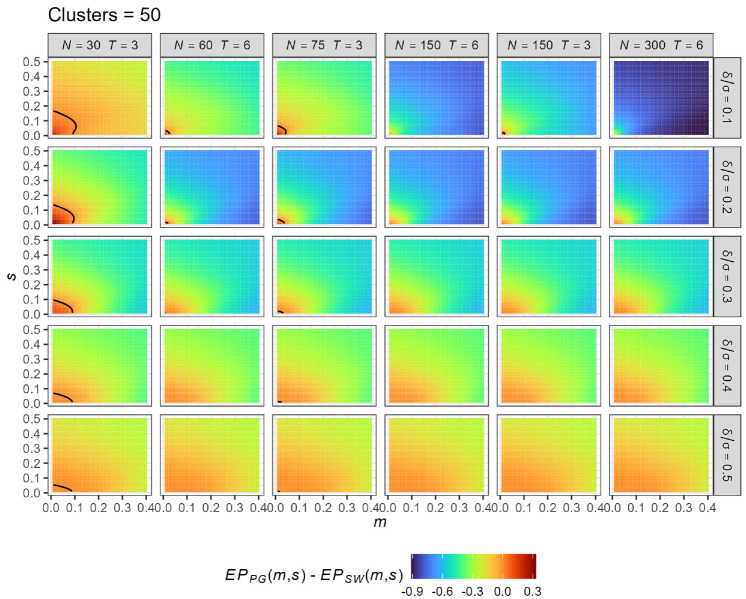
Comparison of the expected power (EP) provided by PG-CRT and SW-CRT designs for different values of the truncated Normal prior parameters m and s, faceted by the assumed effect size ( δ/σ) and assumed values of N (number of participants per cluster) and T (number of time periods in the SW-CRT design). The black curves indicate the point at which the EP is equal for the two designs. Sub-plots without a black curve indicate negative values within the entire region. All results here assume that C=50.

To make the comparison fair, we assume each cluster provides a common number of measurements, setting n=N and n=N/T (for specified N) in the PG- and SW-CRT designs, respectively. We then provide a comparison of the EP for various combinations of the design parameters. Figure 4 assumes C=50; results, shown to be similar, for other values of C are given in Supplementary File 2. A black curve is added to each sub-panel to indicate the (m,s)-contour across which the two designs have equal EP.

We observe that the PG-CRT is better only for very small ICC and very small variance. The maximal values of m and s at which the PG-CRT has larger EP across Figure 4 are m=0.105 and s=0.163; both occurring when N=30 and T=3. As the variance of the prior ( $s^{2})$ increases, the prior places a larger likelihood on a high ICC, which leads to the SW becoming more efficient, even when m≈0. Of 33 reviewed Health Technology Assessment trials that reported assumed ICC values (see Supplementary File 1), 90% of these trials assumed an ICC below 0.105. Thus, whether a PG-CRT design was more efficient than an SW-CRT in practice could heavily depend on the ICC’s uncertainty.

Observe also that the results are sensitive to the values of N and T. Specifically, the region in which the SW-CRT has larger EP increases in size as (i) N is increased for fixed T and δ/σ and (ii) T is increased for fixed N and δ/σ. The pattern as δ/σ is increased for fixed N and T is more complex; though most often increasing the standardized effect size leads to more comparable performance between the two designs, as both transition towards a very high EP.

## Discussion

The significance of the ICC to sample size determination and the challenges associated with pre-specification at the design stage have long been discussed in literature.^35–37^ Our review of Health Technology Assessment reports highlights this further (see Supplementary File 1), a finding consistent with similar review.^11,12^ Motivated by this problem, we therefore presented the detailed calculations required to take a hybrid approach to sample size calculation that allows for direct incorporation of uncertainty on the ICC and the SD. This approach may be advantageous in circumstances where obtaining an accurate ICC estimate during the design stage is problematic, and is more consistent with CONSORT guidance on accounting for ICC uncertainty. Like others have identified in an individually randomized setting,^38,39^ we demonstrated the monotonic relationship between the clusters (sample size) and the EP; thus, an increase in sample size increases the EP, and sample size calculation under a hybrid framework for a CRT functions very similarly to the more familiar frequentist approach. We went on to present a comparison between PG- and SW-CRTs when using this approach. The findings showed that the SW-CRT is more efficient when there is higher uncertainty in the ICC (s≥0.16), even for a small modal ICC assumption (m≤0.1).

Like Kunzmann et al.,^15^ we argue for the control of the EP in designing and determining the sample size of a trial under the hybrid framework since it typically takes values more comparative to the frequentist power. It may in some cases also result in lower required sample sizes, and thus could be deemed an efficient and cost-effective trial design tool, considering the routine high cost of CRTs. In this context, expert opinion could be used to develop appropriate priors, or methodology such as that presented by Turner et al.^17^ could be used to form an informative prior distribution. We discourage the use of uninformative priors such as the uniform distribution since they can be informative in some settings. Having observed from the review of Health Technology Assessment trials, as was also found by Offorha et al.,^12^ that ICCs in health services research are typically small ( ≤ 0.1), a uniform prior that places equal weight on the plausible values of the ICC might not be ideal. A corollary to this is that all priors are inherently subjective and possible misspecification cannot be overlooked. Of course, parameter misspecification is also a problem in frequentist design, and effective prior construction may be reasonably anticipated to mitigate the problem of under- or over-powering on average compared to choosing specific parameter values to assume.

We agree with Hemming et al.^40^ that one design cannot be a panacea to all of the issues and complexities of CRTs. Although sample size, the measure of efficiency in this article, is a key determinant of cost and the probability of detecting a significant effect,^15,41^ the choice of design to use in a particular context must take into consideration a wide array of factors such as the primary objective of the trial. In this sense, this article’s focus on efficiency of a CRT design through the required sample size only is a substantial simplification of choosing an optimal design in practice. Nonetheless, given that cost (an essential consideration in a design choice)^42^ is a function of sample size, we do believe that the significance of our comparison of PG-CRT and SW-CRT under uncertainty should not be downplayed. Such comparisons have previously been conducted by Baio et al.,^42^ Hemming et al.,^43^ and Woertman et al.^21^ in frameworks that did not account for uncertainty in key parameters for sample size calculation.

Our selection of the Health Technology Assessment report (typically more extensive and longer than a clinical journal article) was premised on the fact that it generally serves as a basis for policy implications/recommendations, evidence reviews, technology acquisition, and should arguably represent the upper-end of quality of trial reports.^44^ Therefore, the poor reporting and justification for the assumed ICCs and SD, and lack of adherence to the CONSORT guidelines of making consideration for uncertainties around these parameters was a disappointing finding. This may lead to problems in practice, as CRT sample sizes can be highly sensitive to the choice of these key design parameters. However, we highlight two Health Technology Assessment trials^30,45^ that did provide good justification for the assumed ICC. In particular, Campbell et al.^30^ utilized a 95% confidence interval for the ICC based on estimates from a pilot study.

We acknowledge some limitations to our work. First, our review of one UK journal (Health Technology Assessment trials) may not reflect the entirety of reporting standards for CRTs. In addition, the motivating examples used in this article had continuous outcomes and further studies could benefit from extending this approach to binary outcomes (placing a prior on the control arm response rate). We highlight that these comparisons are not applicable to all CRTs as some, such as certain village surveys, do not require an ICC to be specified. The standard Hussey and Hughes model was also assumed, and we limited our focus to cross-sectional SW-CRT. Therefore, conclusions cannot be made on closed-cohort SW designs or for more complex modelling strategies based on our findings. Nonetheless, this approach could be extended by placing priors on the additional parameters required for closed-cohort SW designs or on the autoregressive parameter of a more complex correlation structure.^46,47^

## Conclusion

In all, when incorporating uncertainty in the ICC, the SW-CRT appears to almost always be a more efficient design relative to the PG-CRT. In general, the greater the uncertainty on the ICC, the more powerful the SW-CRT design over the PG-CRT. This is because an SW-CRT is typically less sensitive (i.e. more efficient, with a lower design effect) for higher values of the ICC, owing to its ability to leverage both within and between cluster comparisons. However, it is notable that the region in which the performance between the designs was similar, in terms of the value of m, does correspond for certain N and T to more commonly assumed values for the ICC. Thus, the uncertainty in the ICC, as captured by s, could be a key determinant of which design is more efficient in practice when using a hybrid approach.

## Supplemental Material

sj-pdf-1-ctj-10.1177_17407745221123507 – Supplemental material for A hybrid approach to comparing parallel-group and stepped-wedge cluster-randomized trials with a continuous primary outcome when there is uncertainty in the intra-cluster correlation Click here for additional data file. Supplemental material, sj-pdf-1-ctj-10.1177_17407745221123507 for A hybrid approach to comparing parallel-group and stepped-wedge cluster-randomized trials with a continuous primary outcome when there is uncertainty in the intra-cluster correlation by Samuel K Sarkodie, James MS Wason and Michael J Grayling in Clinical Trials
