# Supplementary material for: A hybrid approach to comparing parallel-group and stepped-wedge cluster-randomized trials with a continuous primary outcome when there is uncertainty in the intra-cluster correlation
Source: Clin Trials. 2022 Sep 9;20(1):59–70. doi: 10.1177/17407745221123507 (PMC9940131; doi:10.1177/17407745221123507)
Supplement: sj-pdf-1-ctj-10.1177_17407745221123507 – Supplemental material for A hybrid approach to comparing parallel-group and stepped-wedge cluster-randomized trials with a continuous primary outcome when there is uncertainty in the intra-cluster correlation [file sj-pdf-1-ctj-10.1177_17407745221123507.pdf]

Appendix I

Sample Size Calculation in the Frequentist framework

The classical frequentist approach of sample size calculation is to calculate the sample size for an individually randomised trial (IRT) and multiply it by a design effect to account for clustering. The formula for calculating an IRT is given as;

$$n = \frac{2\left(z_{1-\alpha} + z_{1-\beta}\right)^2 \sigma^2}{\delta^2}$$

Note that this formula is for a single arm and must be multiplied by 2 for the total sample size required by a two-arm trial. Here, δ is the effect at which the study is powered at level $1-\beta$ and α is the desired type-I error-rate, achieved by rejecting H_0 when $Z > z_{1-\alpha}$.

The design effects for PG-CRT and cross-sectional SW-CRT are as follows:

$$DE_{PG} = \{1 + (N-1)\rho\}$$

$$DE_{SW} = \frac{1 + \rho(tn + n - 1)}{1 + \rho\left(\frac{tn}{2} + n - 1\right)} \times \frac{3(1-\rho)}{2\left(t - \frac{1}{t}\right)}$$

where ρ is the ICC; N is the sample per cluster for the PG design, n the sample size per cluster-period for the SW design; t is the number of steps (one less than the number of time periods).

Sensitivity analysis: Robustness of Trials designed within the hybrid framework to prior misspecification

We conduct a sensitivity analysis to assess how misspecification of the assumed frequentist ICC and hybrid prior at the design stage impacts the sample sizes in both frameworks. To illustrate this, we first assume that the parameters from our motivating examples are the true values of the ICC (PG: $\rho = 0.1$, SW: $\rho = 0.2$). We then assess how the frequentist and trials designed within the hybrid framework perform if the assumed values (prior modal value in the hybrid case) are greater/less than the true values. The Truncated Normal distribution will be used in the hybrid framework to allow the ICC prior mean to take on values that are equivalent to the frequentist's misspecified values. In terms of the prior variance, we employ a quantile approach such that (a) all the distribution of the misspecified prior is above/below the true ICC value (Q4), (b) 75% of the distribution is above/below the true ICC value (Q3), (c) 50% of the distribution is above/below the true ICC value (Q2), and (d) 25% of the distribution is above/below the true ICC value (Q1). Here, our emphasis is on the ICC since the SD can more easily be accurately obtained through a pilot trial. We present this sensitivity analysis in Table 3 and show a plot of the assumed priors in Figure 4.

In the PG-CRT, when priors are misspecifed such that all the distribution is either above or below the true ICC, the hybrid framework requires a smaller sample size than the frequentist framework. This is advantageous when the mode is larger than the true ICC value, but would result in greater power loss when the mode is smaller the true ICC compared to the frequentist framework. For the remaining hybrid priors, the magnitude of the increase or decrease in sample size compared to the frequentist approach is highly dependent on the percentage of the distribution that is above or below the true ICC value. For example, for a misspecified ICC prior with mean 0.05, 506 participants are required when 75% of the distribution is below the true ICC value, whereas 1056 participants are required to achieve the same desired EP when 25% of the prior is below the true ICC value. The key message from this is that choosing the variance of the prior for the ICC in the case of a PG-CRT would be critical; it could rescue significant power compared to misspecification in the frequentist setting, or result it an even larger waste of resources.

With respect to the SW-CRT, the value of the variance is seemingly less critical, and provided a large variance is avoided (Q1) the study sample size would not be far from that truly required. Again, this is a consequence of the SW design's robustness across possible values of the ICC.

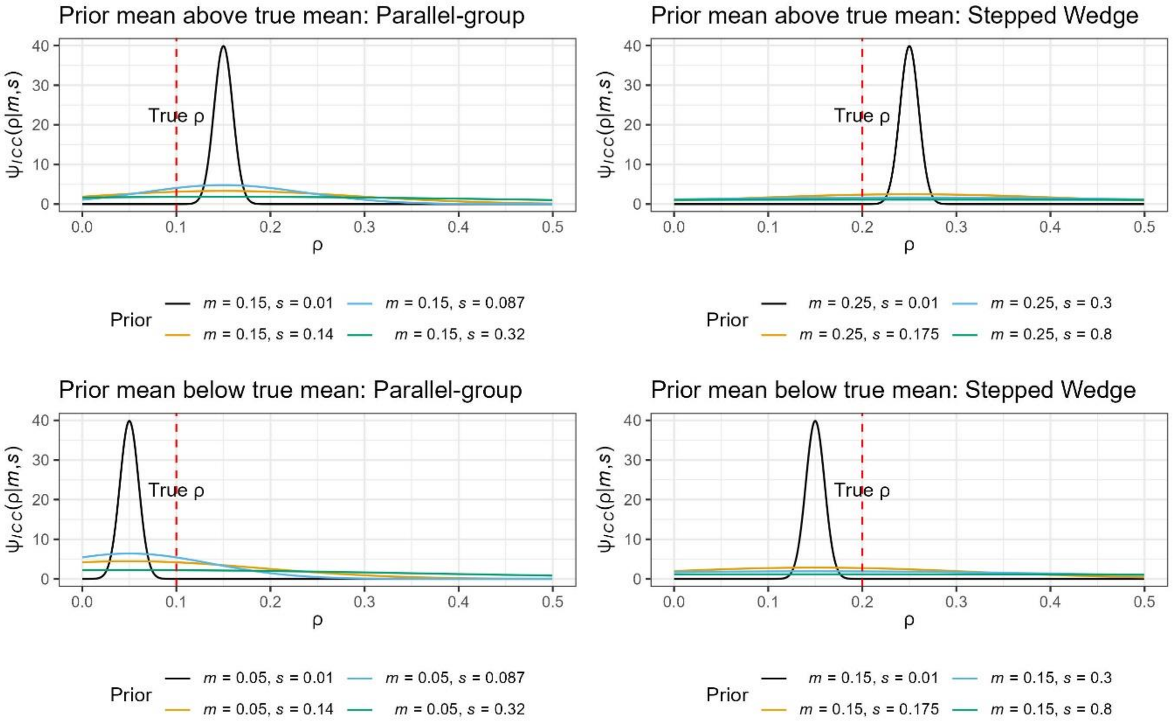

Figure S1: Truncated Normal prior showing the spread of the assumed ICC misspecifications from the true ICC values.

Table S1: Sensitivity analysis of priors for example parameters motivated by Surr et al. (PG-CRT) and O’Grady et al. (SW-CRT); parameter misspecifications. Recall that the true required sample size for the PG-CRT example is 550 participants, while for the SW-CRT it is 3960 participants.

	**Frequentist framework**			**Hybrid framework**		
**Example**	**Parameters**	**assumptions**	**Sample Size**	**Parameters**	**Prior 1: $\boldsymbol{\rho}$ ($\boldsymbol{\psi_2}$)**	**Sample size**
PG-CRT<br>(Surr et al).	$\alpha = 0.025$<br>$\beta = 0.1$<br>$N = 11$<br>$\delta = 3$<br>$\sigma = 7.5$	$\rho = 0.05$	396	$\alpha = 0.025$<br>$\beta = 0.1$<br>$N = 11$<br>$\delta = 3$<br>$\sigma = 7.5$	$TN(0,1, 0.05, 0.010)^{Q4}$	385
					$TN(0,1, 0.05, 0.087)^{Q3}$	506
					$TN(0,1, 0.05, 0.140)^{Q2}$	627
					$TN(0,1, 0.05, 0.320)^{Q1}$	1056
		$\rho = 0.15$	660		$TN(0,1, 0.15, 0.010)^{Q4}$	649
					$TN(0,1, 0.15, 0.087)^{Q3}$	693
					$TN(0,1, 0.15, 0.140)^{Q2}$	781
					$TN(0,1, 0.15, 0.320)^{Q1}$	1177
SW-CRT<br>(O’Grady et al.)	$\alpha = 0.005$<br>$\beta = 0.2$<br>$n = 132$<br>$C = 30$<br>$T = 7$<br>$\delta = 0.028$<br>$\sigma = 0.426$	$\rho = 0.25$	3696	$\alpha = 0.005$<br>$\beta = 0.2$<br>$n = 132$<br>$C = 30$<br>$T = 7$<br>$\delta = 0.028$<br>$\sigma = 0.426$	$TN(0,1, 0.25, 0.010)^{Q4}$	3690
					$TN(0,1, 0.25, 0.175)^{Q3}$	3570
					$TN(0,1, 0.25, 0.300)^{Q2}$	3270
					$TN(0,1, 0.25, 0.800)^{Q1}$	2760
		$\rho = 0.15$	4090		$TN(0,1, 0.15, 0.010)^{Q4}$	4170
					$TN(0,1, 0.15, 0.175)^{Q3}$	3870
					$TN(0,1, 0.15, 0.300)^{Q2}$	3480
					$TN(0,1, 0.15, 0.800)^{Q1}$	2820

Supplementary File 1 – ICC Review from the Health Technology Assessment (HTA) Trials

Paper	Reviewer	Is it a CRT?	What was the justification for the assumed effect (if it's a power-based sample size calculation)?	What was the justification for the assumed SD (if it's a continuous primary outcome)?	What was the assumed value of the ICC?	What was their justification for the chosen ICC/CV?	What was the observed ICC?	Notes
1	SS	Yes	Based on moderate standardized effect of 0.4	Assumed it would be similar to a recently completed trial - citation given for this trial	0.1	Conservative value - cited previous trial	<= 0.058	
2	MG	Yes	None given	NA - binary outcome	Not given	NA	Not given	
3	JW	No	NA	NA	NA	NA	NA	Pilot trial - they calculate the ICC and use it in an example sample size calculation for the main trial. They acknowledge the estimated ICC from the pilot will be very imprecise
4	SS	Yes	None given	NA - binary outcome	0.05	"Power would be higher if the ICC was lower"	<= 0.03	
5	MG	No	NA	NA	NA	NA	NA	Feasibility trial. Note they were ultimately unable to estimate the ICC due to small number of clusters
6	JW	Yes	NA - precision based sample size calculation	NA - binary outcome	0.05	Patterns from previous studies - use moderate value	0.03	
7	SS	Yes	Assumed MCID	From their pilot study	0.1	Unclear	<= 0.05	
8	MG	No	NA	NA	NA	NA	NA	

9	JW	Yes	None given	NA - binary outcome	NA - used coefficient of variation	Two previous studies that they provided citations for	NA - reported coefficient of variation	
10	SS	Yes	Based on what was feasible according to a published Cochrane review	NA - binary outcome	0.05	"Conservative"	0.24 Certainly acknowledged it was larger than anticipated	Assumed conservative value of ICC=0.05, but trial was powered with an ICC of 0.1
11	MG	Yes	None given	None given	0.5	None given	Not given	
12	JW	No	This is a pilot study that was powered to estimate the participation rate (precision of confidence interval of participation rate); number of clusters based on recruiting planned number of patients in funding period	NA - pilot study	0.047	"Using 2010–11 data supplied specifically for this study by the NACR audit team (6272 patients; 119 cardiac rehabilitation teams)," 95% CI given as 0.034 to 0.062	Not given for clinical outcome (high values reported for comparison of self-report measures vs healthcare records)	Recommends using 0.047 (0.034 to 0.062) for planning definitive trial
13	SS	Yes	Based on power calculation to detect a difference of 0.45 standard deviations	From their pilot study	0.062	estimated from the pilot study	0.021	Observed ICC = 0.021 for service level and 0.625 for repeated measure
14	MG	Yes	None given - some indication it may have been effect size based	Previous study that's cited	0.05	Conservative value based on previous cited study	0.139 - note power will have been affected	
15	JW	Yes	0.25 'z-scores' - presumably SD=1. Based on clinically meaningful weight change in children	Standardised outcome - not really given	0.04	"Analysis of data from a previous study" - 0.04 was the maximum limit of the 95% CI	0.021	ICC given with 95% CI for 'unadjusted', 'adjusted' and 'partially adjusted' - 0.0211 (0.0074 to 0.0590) for unadjusted; 0.0193 (0.0063 to 0.0577) adjusted for treatment arm, 0.0858 (0.0501 to 0.1432) for partially adjusted, 0.0805

								(0.0441 to 0.1423) for fully adjusted
16	SS	No	NA	NA	NA	NA	NA	
17	MG	No	NA	NA	NA	NA	NA	Not a CRT
18	JW	Yes	Clinically worthwhile' effect	None provided	0.05	Common in diabetes care'	"ICC from complete case model at 24 months is 0.005. If centre is excluded from the model (as a fixed effect), the ICC is 0.08."	
19	SS	Yes	Assumed MCID	conservative value- from a previous study	0.002	Conservative value - adjusted from previous trial		
20	MG	Yes	None given	NA-binary outcome	0.01	Conservative value	Not given	Note that the ICC was monitored at interim analyses
21	JW	Yes	Not given	NA	0.03	Conservative based on a systematic review	0.026	
22	SS	Yes	None given	None given	0.05	None given	-0.05	
23	MG	Yes	None given	NA-binary outcome	0.026	Cited paper that suggested this value for a similar outcome	Unclear - they've reported so many ICCs	
24	JW	No	NA	NA	NA	NA	NA	Not a CRT
25	SS	Yes	None	None given	0.01	None given	0.002	
26	MG	No	NA	NA	NA	NA	NA	Not a CRT
27	JW	Yes	Clinically meaningful effect	Previous trials of similar interventions	0.4	Conservative assumption based on previous CRTs	0.09	Gives higher estimated ICC for baseline measurement, and 0.09 for follow-up adjusted for baseline etc
28	SS	Yes	Assumed MCID	None given	0.06	Pilot study	0	
29	MG	No	NA	NA	NA	NA	NA	No formal sample size calculation

30	JW	Unsure	None given	NA	0.02	Previous studies	Unable to estimate	This was a feasibility study but sample size was based on definitive study.
31	SS	Yes	None as it was a pilot study	NA	NA	NA	GBS - 0.522; AAYP - 0.419; ESYTC - 0.426	
32	MG	Yes	Previous study	Previous study	0.05	Previous study and previous review work - also give power for several values	0.015 - they note this is lower than planned for	They did a pilot that gave a 95% CI for the ICC
33	JW	No	NA	NA	NA	NA	NA	No sample size section
34	SS	Yes	None	None given	0.01	previous studies	0.009 (Log transformed)	
35	MG	Yes	Moderate effect size	None given	0.1	Based on available database of ICCs as they didn't have any data	0.134 at 12 months and 0.064 at 24 months	
36	JW	No	NA	NA	NA	NA	NA	Feasibility trial, sample size not based on statistical arguments
37	SS	Yes	Based on power calculation to detect a difference of 0.35 standard deviations	Pilot study	0.025	Pilot study		
38	MG	Yes	Clinical relevance - citation given	None given	0.05	Based on research showing this was a conservative choice	Adjusted is 0.027	
39	JW	Yes	Standardised effect 'medium effect'	Standardised outcome - not given	0.11	Not given	0.005	
40	SS	Yes	None given	None given	0.05	Conservative	>=0.053	
41	MG	No	NA	NA	NA	NA	NA	Split plot design
42	JW	Yes	Not given, 'moderate effect' on standardised difference provided	Standardised outcome - not given	0.08	Based on audit data	0.057	

43	SS	Yes	None given	None given	0.05	conservative		
44	MG	No	NA	NA	NA	NA	NA	Not a trial
45	JW	Yes	Not given	NA	0.03	Based on 31 studies in the area	0.038 (0 to 0.097), 0.061 (0 to 0.137), 0.142 (0.010 to 0.275) in the three arms	Sample size was increased based on ICC estimated at interim
46	SS	Yes	MCID	None given	0.006	Based on a 6-month EPDS scores by GP practice	0.037	
47	MG	No	NA	NA	NA	NA	NA	Not a trial
48	JW	No	NA	NA	NA	NA	NA	
49	SS	No	NA	NA	NA	NA	NA	
50	MG	No	NA	NA	NA	NA	NA	Not a trial
51	JW	Yes	Not given	NA	0.05	Obtained from pre-intervention survey	0.009	
52	SS	No	NA	NA	NA	NA	NA	
53	MG	Yes	Smaller than that likely to be considered clinically meaningful	Previous study that's cited	0.01	Previous study that's cited	Reported by arm, but all <0.006	
54	JW	No	NA	NA	NA	NA	NA	Not a CRT

Supplementary File 2

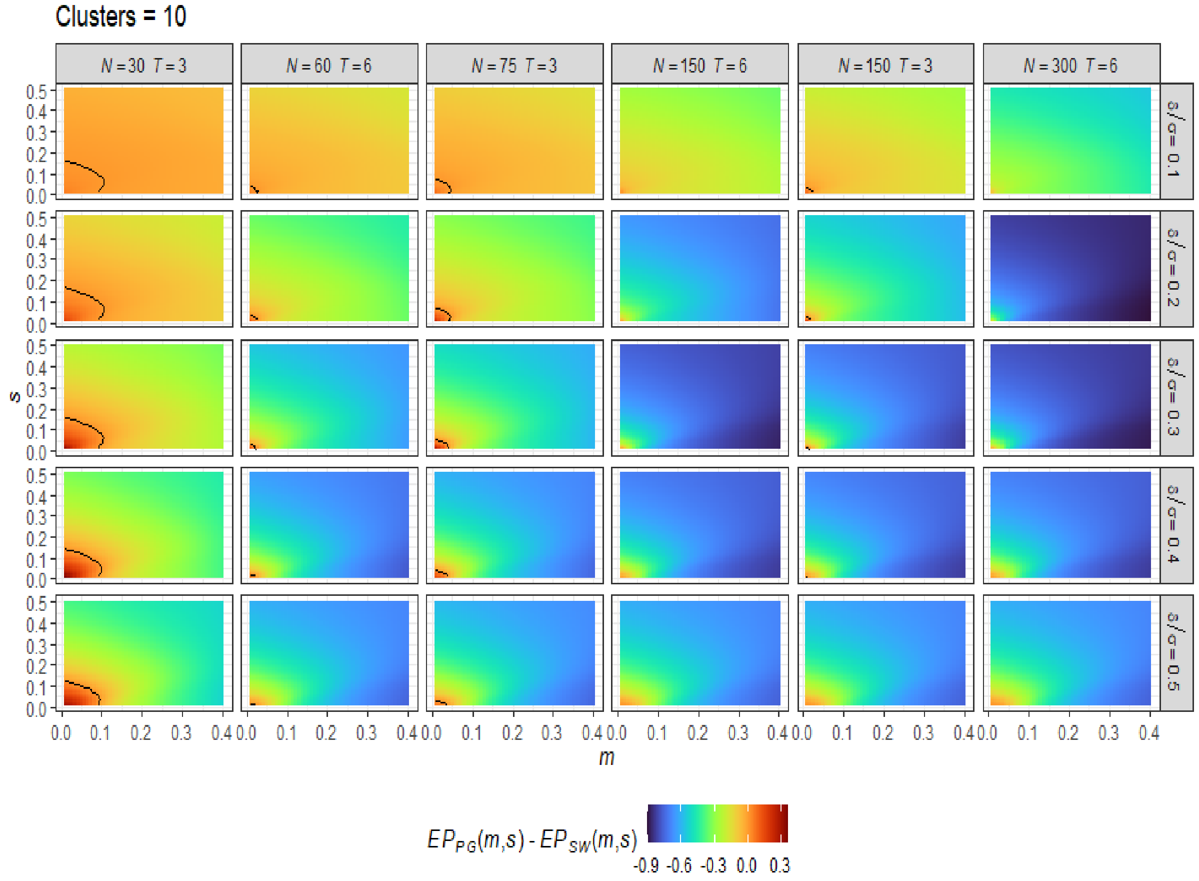

Clusters = 25

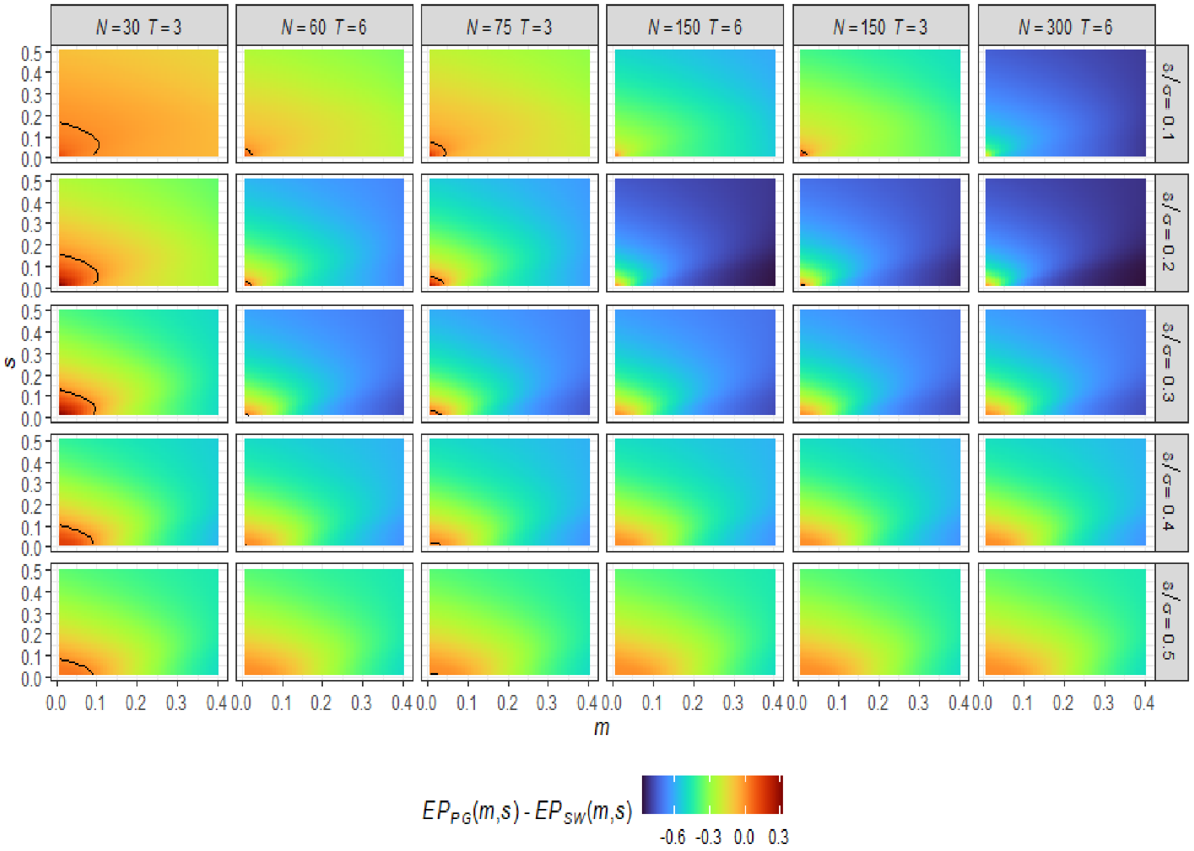

N = 30 T = 3
N = 60 T = 6
N = 75 T = 3
N = 150 T = 6
N = 150 T = 3
N = 300 T = 6
δ/σ = 0.1
δ/σ = 0.2
δ/σ = 0.3
δ/σ = 0.4
δ/σ = 0.5
s
m
0.0 0.1 0.2 0.3 0.4 0.5
0.0 0.1 0.2 0.3 0.4
$EP_{PG}(m,s) - EP_{SW}(m,s)$
-0.6 -0.3 0.0 0.3

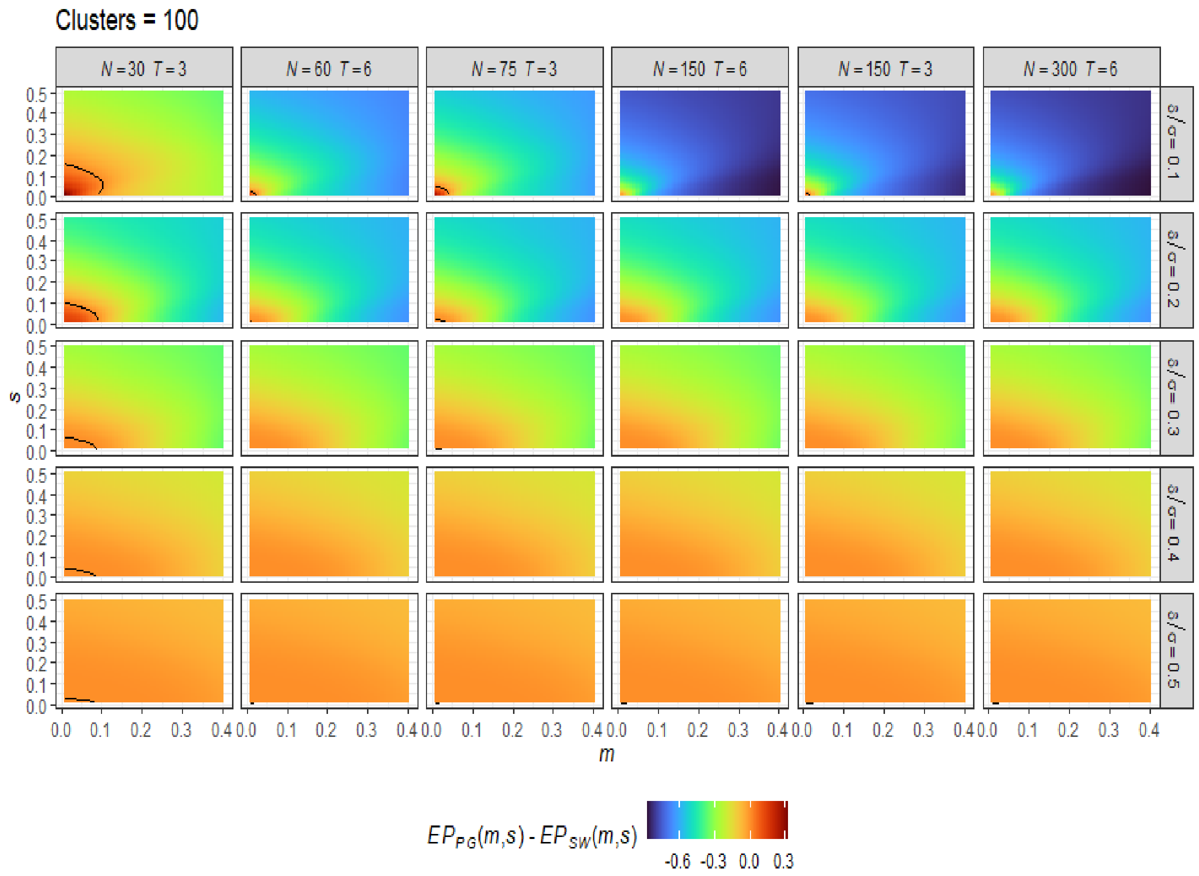
Clusters = 100
N = 30 T = 3
N = 60 T = 6
N = 75 T = 3
N = 150 T = 6
N = 150 T = 3
N = 300 T = 6
δ/σ = 0.1
δ/σ = 0.2
δ/σ = 0.3
δ/σ = 0.4
δ/σ = 0.5
s
m
EP_PG(m,s) - EP_SW(m,s)
-0.6 -0.3 0.0 0.3
